# Serum lipid and bone metabolism effects of Toremifene vs. Letrozole as adjuvant therapy for postmenopausal early breast cancer patients: results of a multicenter open randomized study

**DOI:** 10.1007/s00280-017-3491-6

**Published:** 2017-12-01

**Authors:** Tadahiko Shien, Hiroyoshi Doihara, Nobuaki Sato, Keisei Anan, Kansei Komaki, Keisuke Miyauchi, Yasuhiro Yanagita, Tomomi Fujisawa, Shoshu Mitsuyama, Chizuko Kanbayashi, Mikihiro Kusama, Morihiko Kimura, Hiromitsu Jinno, Muneaki Sano, Tadashi Ikeda

**Affiliations:** 1 Department of Breast and Endocrine Surgery, Okayama University Hospital, 2-5-1 Shikata-cho, Kita-ku, Okayama, 700-8558 Japan; 2 Niigata Cancer Center Hospital, Niigata, Japan; 3 Kitakyushu Municipal Medical Center, Fukuoka, Japan; 4 Breastopia Miyazaki Hospital, Miyazaki, Japan; 5 Miyauchi Clinic, Hyogo, Japan; 6 Gunma Prefectural Cancer Center, Gunma, Japan; 7 Shinjuku Breast Center, Tokyo, Japan; 8 Ota General Hospital, Gunma, Japan; 9 Teikyo University School of Medicine, Tokyo, Japan; 10 Niigata Breast Examination Center, Niigata, Japan

**Keywords:** Adjuvant therapy, Endocrine therapy, Postmenopausal, Lipid metabolism, Bone metabolism

## Abstract

A prospective randomized phase II trial was conducted to evaluate the time course effects of toremifene (TOR) and letrozole (LET), as adjuvant hormone therapy, on serum lipid profiles and bone metabolism in estrogen receptor (ER)-positive, postmenopausal breast cancer patients.Fifty-four postmenopausal breast cancer patients [ER positive, HER2 negative, T1–2, node metastases (*n* = 0–3), M0] who had undergone curative resection were enrolled. They were randomized to receive either TOR 40 mg/day or LET 2.5 mg/day as adjuvant hormone therapy. Serum lipids and bone markers were measured prior to, and again at 6, 12, and 24 months after initiation of treatment. Changes in serum lipids and bone markers were compared. Serum levels of total cholesterol (TC) and low-density lipoprotein cholesterol (LDL-C) were decreased compared with the baseline values at 6 months in 6.5 and 14.0% of patients, respectively, receiving TOR. Lipid levels did not change in patients administered LET. Significant differences were observed in TC and LDL-C between the two groups at 12 and 24 months. In the TOR group, serum bone-specific alkaline phosphatase (BAP) was decreased by 25.0% at 12 months, and serum cross-linked N-telopeptide of type-I collagen (NTx) was decreased by 13.6% at 6 months, and these reductions were maintained for at least 24 months. In contrast, in the LET group, serum BAP did not change and NTx was increased by 16.0% at 6 months and by 18.6% at 24 months, as compared with the baseline.TOR and LET exert different effects on serum lipid profiles and bone metabolism markers. The effects of TOR, as adjuvant hormone therapy, on both lipids and bone metabolism in postmenopausal breast cancer patients are superior to those of LET.

## Introduction

Selective estrogen receptor modulators (SERMs) and aromatase inhibitors (AI) are standard adjuvant hormone therapies for estrogen receptor (ER) positive breast cancer patients. Several SERM and AI are currently available. To prevent recurrence, patients are treated with one of these drugs for at least 5 years after curative surgery. However, selecting the most appropriate drug for an individual patient is difficult due to lack of data, especially regarding adverse effects.

The influences on lipid and bone metabolism are among the most important adverse effects of hormone therapy. These side effects must be taken into consideration when prescribing SERMs and AI. Toremifene (TOR), a SERM, has prognostic effects and a safety profile similar to those of tamoxifen (TAM) [1, 2]. Moreover, Harvey et al. reported that the risks of stroke, pulmonary embolism, and cataract may be lower with TOR than with TAM and that the risks of pulmonary embolism and deep vein thrombosis are lower than with raloxifene [3].

Letrozole (LET) is a non-steroidal AI. When LET or TAM was administered as adjuvant hormone therapy for ER positive postmenopausal breast cancer patients, LET reflected an 18% reduction in the risk of a disease-free survival event (hazard ratio 0.82; 95% CI 0.71–0.95; *P* = 0.007). The 5-year disease-free survival estimates were 84.0% in LET and 81.1% in TAM, though differences in the hazard ratios for overall survival did not reach statistical significance. Patients on TAM experienced more thromboembolic events, endometrial pathology, hot flashes, night sweats, and vaginal bleeding. Patients receiving LET experienced more bone fractures, arthralgia, low-grade hypercholesterolemia, and cardiovascular events other than ischemia and cardiac failure [4].

We previously reported the results of two trials. In the Multi 01 trial, two SERM, TAM and TOR, were found to exert different effects on lipid metabolism, profiles with TOR producing better results than TAM [5]. Moreover, TOR provided better effects than anastrozole (ANA), an AI, in terms of lipid profiles and bone metabolism in postmenopausal females with early breast cancer in the Multi 02 study [6]. LET, however, exerts effects different from those of ANA. Ideally, adjuvant therapy with these hormonal agents should be tailored to individual patients.

In this trial, we examined the differences between TOR and LET in terms of their influences on lipid profiles and bone metabolism, by conducting a prospective randomized phase II study. Our aim was to collect important data relevant to quality of life for patients receiving hormone therapy.

## Patients and methods

### Study design and drug administration

This was a multicenter open randomized study. Study endpoints were the time course effects of TOR and LET on serum lipid profiles and bone metabolism in ER positive, postmenopausal breast cancer patients receiving these adjuvant hormonal therapies. The protocol was approved by the research ethics committee of each participating institution. Patients were provided written information about the study and gave written informed consent prior to enrollment. Randomization was performed using the minimization method, and the arms were balanced with regard to institution. All patients were randomized to TOR or LET. In the TOR and LET arms, TOR (40 mg/day) and LET (2.5 mg/day) were administered for a minimum of 2 years, respectively. During the study, other treatments for breast cancer were prohibited except for radiotherapy after breast-conserving surgery.

### Eligibility and exclusion criteria

Postmenopausal women with Stage I–II breast cancer were eligible for this study. All patients had to have undergone curative surgery, with histological examination confirming immunohistologically ER positive and HER2 negative breast cancer with involvement of 0–3 axillary nodes. Other eligibility criteria were a World Health Organization (WHO) performance status of 0–1, adequate bone marrow and liver and kidney functions, and no evidence of metastasis. Patients who had received previous systemic treatments for breast cancer and/or other drugs which might influence serum lipid profiles and bone metabolism were excluded. Patients who had been diagnosed with osteoporosis and administered hormone replacement therapy were not eligible. All enrolled patients had signed the aforementioned consent form prior to enrollment.

### Patient assessment

#### Lipid analysis

The first blood sample was collected before breakfast. In the afternoon, blood was collected before lunch, with an interval of at least 3 h from breakfast until blood collection. Immediately after blood collection, serum was isolated. The triglyceride (TG), total cholesterol (TC), HDL-C, and LDL-C levels were measured before starting hormonal agent administration and then again 6, 12, and 24 months after commencement of the study medication. The assays were performed at SRL Inc. (Tokyo). The TG, TC and LDL-C levels were measured employing enzymatic assays, HDL-C was measured by a direct method. Abnormal levels of TC, LDL-C, HDL-C, and TG were established as > 220, > 40, < 40, and > 150 mg/dl, based on the reference values, as described in the guidelines for hyperlipidemia treatment in Japan [7]. Patients persistently showing a TC level of 350 mg/dl or higher and an LDL-C level above 200 mg/dl for at least 1 year during treatment were regarded as drop-outs, and agents to treat hyperlipidemia were administered. Diet/exercise therapies were prescribed and explained, if necessary.

#### Bone metabolism analysis

The levels of bone-specific alkaline phosphatase (BAP), a bone formation marker, N-telopeptide of type-I collagen (NTX), a bone resorption marker, and Homocysteine (Hcy), a bone metabolic marker, were measured before administration of the hormonal agent and then again 6, 12, and 24 months after commencement of the study medication. BAP was measured employing an enzyme immunoassay, NTX by ELISA and Hcy by high performance liquid chromatography at SRL Inc., Tokyo.

### Statistical analysis

Based on the data obtained in our previous study showing serum HDL-C to be increased by 13.4 ± 11.3 (mean ± SD) after a 1-year TOR administration period, and on the assumption that the serum HDL-C level would not be changed by LET administration, it was determined that having at least 15 subjects in each group would provide 80% power at the 5% significance level to detect a difference between the two groups. To allow for drop-outs, the sample size was increased to 30 in each treatment arm. Because the TG, TC, HDL-C, and LDL-C values were essentially normal, the mean was the preferred summary statistic. Serial changes in these variables in each group were analyzed using a paired *t* test. The serial changes in BAP, NTX and Hcy were analyzed employing the Wilcoxon signed ranks test. The SAS software program was used for all analyses.

## Results

**Fig. 1:**
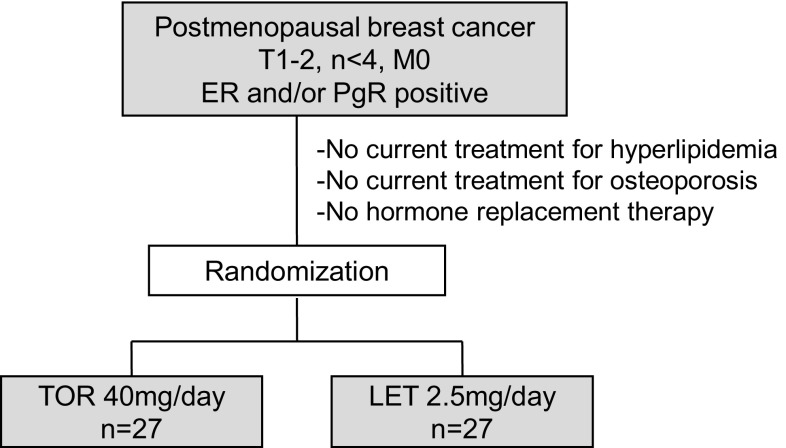
Study design

The 54 patients enrolled in this study were randomly assigned to the TOR group (*n* = 27) or the LET group (*n* = 27) for adjuvant therapy (Fig. 1). The median age of the patients in the TOR group was 61 years and that in the LET group was 65 years (N.S.). Height, body weight, and BMI did not differ significantly between the two groups (Table 1). 24 in LET and 25 in TRE group patients could continue taking drugs at least 2 years. One patient in LET group stopped at 6 months and other four patients could not continue for 6 months because of non-hematological side effects of LET and TRE, respectively.

**Table 1:** Baseline characteristics of patients

	TOR (*n* = 27)	LET (*n* = 27)	*P* value
Age (median, years)	60.5 (46–75)	65 (46–74)	ns
Height (cm)	153 (145–167)	153 (139–164)	ns
Weight (kg)	56.5 (45–68)	53 (41–73)	ns
BMI (kg/m^2^)	23.3 (20.0–28.5)	22.0 (18.8–32.8)	ns

### Effects on lipid metabolism

**Fig. 2:**
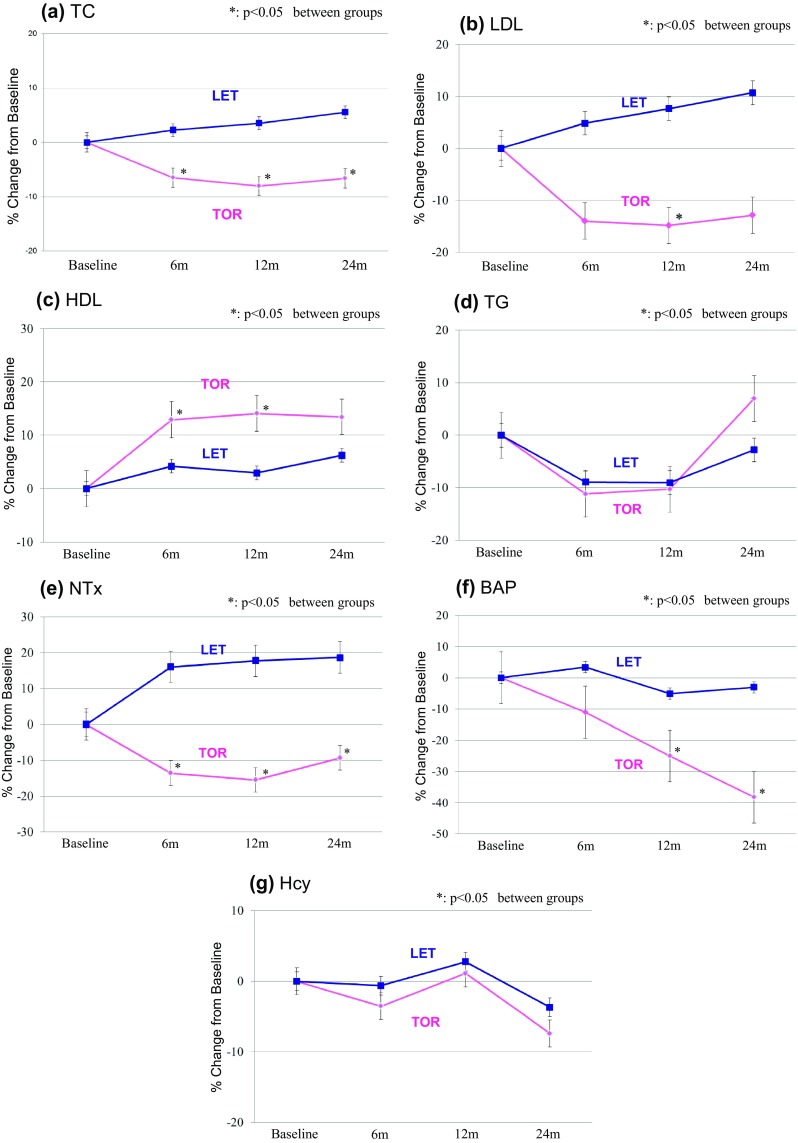
Changes from baseline in lipids and bone profiles at 6, 12, and 24 months

In the TOR group, LDL and TC were significantly decreased at 6 months as compared with the baseline value (*P* < 0.0005) (Tables 2, 3; Fig. 2b). HDL was significantly increased at 6, 12, and 24 months in the TOR group (*P* < 0.0005) (Fig. 2c), while these markers were essentially unchanged in the LET group. Comparison of the TOR and LET groups revealed the percent changes in LDL and TC values to differ significantly between the two groups at 12 and 24 months (*P* < 0.005). There was no significant TG change in either group (Fig. 2).

**Table 2:** Comparisons of mean values before and at 6,12, and 24 months

TOR					LET			
Before	6 months	12 months	24 months		Before	6 months	12 months	24 months
27	22	24	24	*n*	27	24	21	21
224.2	206.5*	206.3*	204.9*	TC 150–219 (mg/dL)	213.9	219.3	221.0	227.6
138.7	124.9	129.4	140.8	TG 50–149 (mg/dL)	131.3	105.5*	101.7	104.0
57.9	64.5*	65.4*	64.4*	HDL 40–96 (mg/dL)	58.3	60.4	60.1	62.4
133.4	112.8*	113.8*	112.7*	LDL 70–139 (mg/dL)	124.2	130.0	131.1	138.0
26.0	22.9*	19.8*	16.4*	BAP 9.6–35.4 (U/L)	26.9	27.8	24.7	26.0
16.3	14.5*	13.5*	15.9	NTx 10.7–24.0 (nMBCE/L)	15.6	17.0	17.2	17.6
10.1	9.2	9.6	8.8	Hcy	9.8	9.2	9.8	8.9

*P* value compared with “Before” *P < 0.05

**Table 3:**
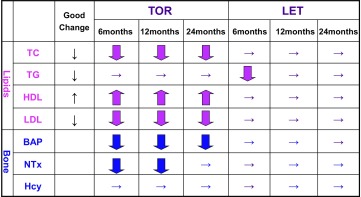
Comparisons of mean values before and at 6, 12, and 24 months, summary

### Effects on bone metabolism

In the TOR group only, BAP was significantly decreased at 6, 12, and 24 months and NTx was significantly decreased at 6 and 12 months (*P* < 0.05)(Tables 2, 3). Comparison of the TOR and LET groups revealed percent changes in the values of all markers to differ significantly between the two at 6, 12, and 24 months (*P* < 0.05), the only exception being BAP at 6 months. Hcy levels did not differ significantly between the two groups (Fig. 2).

## Discussion

AI as adjuvant hormone therapy are considered to be superior to TAM for postmenopausal breast cancer patients. BIG 1–98 reported better disease free survival in breast cancer patients taking LET for 5 years, though there was no significant difference in overall survival between LET and TAM [8]. Considering the long-term effects of hormonal agents, we should take both prognostic and adverse effects into consideration when selecting the most appropriate hormonal agent for an individual patient. Differences in the adverse effects of hormonal agents must be taken into account when deciding on a treatment strategy.

From the results of the FBCC [1] and IBCSG [2] trials, TOR as a drug for adjuvant hormone therapy has prognostic effects and safety features similar to those of TAM. Differences from TAM include fewer thrombotic events, a lower estrogenic effect on genital organs, and better effects on serum lipids with TOR. We have previously shown that TOR is superior to TAM in terms of the serum lipid profile [5] and that TOR provides better effects than ANA in terms of lipid profiles and bone metabolism in postmenopausal females with early breast cancer [6].

### Lipid metabolism

TOR administration was associated with a significantly better lipid metabolism profile than LET in this study. Mega trials of AI as adjuvant treatment identified significant hyperlipidemia and cardiovascular disease. SERM therapies, including TAM, have been shown to exert beneficial effects on serum lipid profiles [9].

Numerous studies have shown that hypercholesterolemia is an important risk factor for coronary heart disease (CHD) [10, 11]. A high LDL-C level and a low HDL-C level are important and well-established risk factors for CHD [12, 13]. Moreover, the Copenhagen Male study found that patients with both high TG and low HDL-C were at risk for ischemic heart disease [14]. On the other hand, treating hypercholesterolemia in CHD patients prevents the recurrence of CHD [15]. For breast cancer patients with a CHD history, the adverse influence of adjuvant hormone therapy on lipid metabolism remains a very significant clinical problem.

### Bone metabolism

Bone metabolism is a significant clinical parameter that should be factored into any decisions about adjuvant treatment of postmenopausal women with breast cancer. Fractures have been recognized as major problems for the elderly. The femoral neck fractures associated with osteoporosis have a severe adverse impact on activities of daily living. Moreover, osteoporosis is reportedly related to prognostic worsening in elderly people [16, 17]. Checking bone metabolism parameters is important for determining the optimal adjuvant hormone therapy regimen. The rate of bone fracture in patients receiving adjuvant AI is approximately 1.4–1.5 times that with adjuvant TAM [18–21]. A previous study focusing on deterioration of bone metabolism found AI to have a more deleterious effect than TAM [22]. In most postmenopausal women, both BAP and NTx increase more than in premenopausal women due to diminished serum estrogen with menopause. SERMs have effects on both bone formation and bone resorption. TOR can maintain or slightly increase bone mineral density. In this study, TOR had a significantly more beneficial influence than LET on serum NTx and BAP. Bone turnover decreased after the administration of TOR, while being relatively increased after administration of LET in postmenopausal women with early breast cancer, suggesting that TOR contributes to preventing osteoporosis. Hcy is a known risk factor for heart attacks and strokes. Its measurement may be useful in patients with a family history of CHD but no other known risk factors, such as smoking, high blood pressure, or obesity. Recently, a high Hcy level was reported to be associated with bone fracture and but not with bone mineral density [23, 24]. The authors of that study suggested that Hcy may destroy the structure of collagen. In this study, there was no significant change in the Hcy level in either group.

Recently, FACE trial revealed that LET did not have significantly superior efficacy and safety compared with ANA [25]. The safety profiles of two treatment arms (LET vs. ANA) were similar, osteoporosis (10.3 vs. 9.4%), clinical fractures (9.3 vs. 8.0%), and ischemic heart disease (2.4 vs. 1.5%), respectively. We reported previously TOR provided better effects than ANA in Multi 02 study. ANA had the significant worse effects for lipid and bone metabolism. In this study, LET had similar worse effects in those metabolisms with ANA.

## Conclusion

TOR and LET have different clinical profiles. Clinicians should be aware of these differences when choosing a hormonal agent, especially when the administration of adjuvant treatment is anticipated to be at least 5 years. Compared with LET, TOR exerts beneficial effects on both lipids and bone metabolism. TOR would appear to be the optimal treatment for postmenopausal breast cancer patients, especially the elderly, with CHD, hyperlipidemia, and/or osteoporosis. This study was a small phase II trial and these results had some limitation. Additional study in the patients belonging to big prospective adjuvant trials of endocrine therapy are needed to confirm our findings.

## Abbreviations

TOR: Toremifene
LET: Letrozole
ANA: Anastrozole
CHD: Coronary heart disease
TC: Total cholesterol
TG: Triglyceride
HDL-C: High-density lipoprotein cholesterol
LDL-C: Low-density lipoprotein cholesterol
BAP: Bone-specific alkaline phosphatase
NTx: N-telopeptide of type-I collagen
Hcy: Homocysteine

## References

[1] Holli K, Valavaara R, Blanco G (2000). Safety and efficacy results of a randomized trial comparing adjuvant toremifene and tamoxifen in postmenopausal patients with node-positive breast cancer. Finnish Breast Cancer Group. J Clin Oncol.

[2] Pagani O, Gelber S, Price K (2004). Toremifene and tamoxifen are equally effective for early-stage breast cancer: first results of International Breast Cancer Study Group Trials 12–93 and 14–93. Ann Oncol.

[3] Harvey HA, Kimura M, Hajba A (2006). Toremifene: an evaluation of its safety profile. Breast.

[4] Coates AS, Keshaviah A, Thürlimann B (2007). Five years of letrozole compared with tamoxifen as initial adjuvant therapy for postmenopausal women with endocrine-responsive early breast cancer: update of study BIG 1–98. J Clin Oncol.

[5] Kusama M, Miyauchi K, Aoyama H (2004). Effects of toremifene (TOR) and tamoxifen (TAM) on serum lipids in postmenopausal patients with breast cancer. Breast Cancer Res Treat.

[6] Anan K, Mitsuyama S, Yanagita Y (2011). Effects of toremifene and anastrozole on serum lipids and bone metabolism in postmenopausal females with estrogen receptor-positive breast cancer: the results of a 2-year multicenter open randomized study. Breast Cancer Res Treat.

[7] Japan Atheroclerosis Society (2007) Japan atherosclerosis society guidelines for prevention of atherosclerotic cardiovascular diseases. Jpn Atheloscler Soc 6

[8] Mouridsen H, Giobbie-Hurder A, Goldhirsch A (2009). Letrozole therapy alone or in sequence with tamoxifen in women with breast cancer. N Engl J Med.

[9] Riggs BL, Hartmann LC (2003). Selective estrogen-receptor modulators: mechanisms of action and application to clinical practice. N Engl J Med.

[10] Kannel WB, Castelli WP, Gordon T (1971). Serum cholesterol, lipoproteins, and the risk of coronary heart disease. The Framingham study. Ann Intern Med.

[11] Martin MJ, Hulley SB, Browner WS (1986). Serum cholesterol, blood pressure, and mortality: implications from a cohort of 361, 662 men. Lancet.

[12] Assmann G, Schulte H (1992). Relation of high-density lipoprotein cholesterol and triglycerides to incidence of atherosclerotic coronary artery disease (the PROCAM experience). Prospective Cardiovascular Münster study. Am J Cardiol.

[13] Manninen V, Tenkanen L, Koskinen P (1992). Joint effects of serum triglyceride and LDL cholesterol and HDL cholesterol concentrations on coronary heart disease risk in the Helsinki Heart Study. Implications for treatment. Circulation.

[14] Jeppesen J, Hein HO, Suadicani P (1997). Relation of high TG-low HDL cholesterol and LDL cholesterol to the incidence of ischemic heart disease. An 8-year follow-up in the Copenhagen Male Study. Arterioscler Thromb Vasc Biol.

[15] Smith GD, Pekkanen J (1994). Randomised trial of cholesterol lowering in 4444 patients with coronary heart disease: the Scandinavian Simvastatin Survival Study (4S). Lancet.

[16] Ensrud KE, Thompson DE, Cauley JA (2000). Prevalent vertebral deformities predict mortality and hospitalization in older women with low bone mass. Fracture Intervention Trial Research Group. J Am Geriatr Soc.

[17] Nguyen ND, Center JR, Eisman JA (2007). Bone loss, weight loss, and weight fluctuation predict mortality risk in elderly men and women. J Bone Miner Res.

[18] Coombes RC, Hall E, Gibson LJ (2004). A randomized trial of exemestane after two to three years of tamoxifen therapy in postmenopausal women with primary breast cancer. N Engl J Med.

[19] Howell A, Cuzick J, Baum M (2005). Results of the ATAC (Arimidex, Tamoxifen, Alone or in Combination) trial after completion of 5 years’ adjuvant treatment for breast cancer. Lancet.

[20] Jakesz R, Jonat W, Gnant M (2005). Switching of postmenopausal women with endocrine—responsive early breast cancer to anastrozole after 2 years’ adjuvant tamoxifen: combined results of ABCSG trial 8 and ARNO 95 trial. Lancet.

[21] Thürlimann B, Keshaviah A, Coates AS (2005). A comparison of letrozole and tamoxifen in postmenopausal women with early breast cancer. N Engl J Med.

[22] Aihara T, Suemasu K, Takei H (2010). Effects of exemestane, anastrozole and tamoxifen on bone mineral density and bone turnover markers in postmenopausal early breast cancer patients: results of N—SAS BC 04, the TEAM Japan substudy. Oncology.

[23] McLean RR, Jacques PF, Selhub J (2004). Homocysteine as a predictive factor for hip fracture in older persons. N Engl J Med.

[24] van Meurs JB, Dhonukshe-Rutten RA, Pluijm SM (2004). Homocysteine levels and the risk of osteoporotic fracture. N Engl J Med.

[25] Smith I, Yardley D, Burris H (2017). Comparative efficacy and safety of adjuvant Letrozole versus Anastrozole in postmenopausal patients with hormone receptor-positive, node-positive early breast cancer: final results of the randomized phase III Femara versus Anastrozole Clinical Evaluation (FACE) Trial. J Clin Oncol.

